# Novel methylation-related long non-coding RNA clinical outcome prediction method: the clinical phenotype and immune infiltration research in low-grade gliomas

**DOI:** 10.3389/fonc.2023.1177120

**Published:** 2023-05-09

**Authors:** Youjun Li, Xiaobo Li, Zhengtao Yu

**Affiliations:** Department of Neurosurgery, Affiliated Haikou Hospital of Xiangya School of South University, Haikou, Hainan, China

**Keywords:** low-grade glioma, prognostic model, LncRNA, M1A/M5C/M6A, immune infiltration

## Abstract

**Background:**

Recent studies have suggested that long non-coding RNAs (lncRNAs) may play crucial role in low-grade glioma; however, the underlying mechanisms linking them to epigenetic methylation remain unclear.

**Methods:**

We downloaded expression level data for regulators associated with N1 methyladenosine (m1A), 5-methyladenine (m5C), and N6 methyladenosine (m6A) (M1A/M5C/M6A) methylation from the Cancer Genome Atlas-low-grade glioma (TCGA-LGG) database. We identified the expression patterns of lncRNAs, and selected methylation-related lncRNAs using Pearson correlation coefficient>0.4. Non-negative matrix dimensionality reduction was then used to determine the expression patterns of the methylation-associated lncRNAs. We constructed a weighted gene co-expression network analysis (WGCNA) network to explore the co-expression networks between the two expression patterns. Functional enrichment of the co-expression network was performed to identify biological differences between the expression patterns of different lncRNAs. We also constructed prognostic networks based on the methylation presence in lncRNAs in low-grade gliomas.

**Results:**

We identified 44 regulators by literature review. Using a correlation coefficient greater than 0.4, we identified 2330 lncRNAs, among which 108 lncRNAs with independent prognostic values were further screened using univariate Cox regression at P< 0.05. Functional enrichment of the co-expression networks revealed that regulation of trans-synaptic signaling, modulation of chemical synaptic transmission, calmodulin binding, and SNARE binding were mostly enriched in the blue module. The calcium and CA2 signaling pathways were associated with different methylation-related long non-coding chains. Using the Least Absolute Shrinkage Selector Operator (LASSO) regression analysis, we analyzed a prognostic model containing four lncRNAs. The model’s risk score was 1.12 *AC012063 + 0.74 * AC022382 + 0.32 * AL049712 + 0.16 * GSEC. Gene set variation analysis (GSVA) revealed significant differences in mismatch repair, cell cycle, WNT signaling pathway, NOTCH signaling pathway, Complement and Cascades, and cancer pathways at different GSEC expression levels. Thus, these results suggest that GSEC may be involved in the proliferation and invasion of low-grade glioma, making it a prognostic risk factor for low-grade glioma.

**Conclusion:**

Our analysis identified methylation-related lncRNAs in low-grade gliomas, providing a foundation for further research on lncRNA methylation. We found that GSEC could serve as a candidate methylation marker and a prognostic risk factor for overall survival in low-grade glioma patients. These findings shed light on the underlying mechanisms of low-grade glioma development and may facilitate the development of new treatment strategies.

## Introduction

Gliomas are the most common primary tumors in the human brain and spinal cord. The World Health Organization (WHO) classified the primary central nervous system (CNS) tumors in 2007 using histopathological diagnostic analysis. Gliomas can be classified by cell type into astrocytomas, oligodendrogliomas, neuronal and mixed neuron gliomas, ependymomas, or oligodendrogliomas. Gliomas can also be graded from the least to most aggressive (Grades I to IV), with grades I and II indicating low-grade gliomas and grades III and IV showing high-grade gliomas (1–3). The median survival time is 11.6 years for low-grade gliomas, about three years for patients with grade III gliomas, and 15 months for patients with grade IV gliomas (4). Therefore, it is essential to study the mechanisms mediating the progression and prognosis of glioma.

RNA post-transcriptional modifications, including N6 methyladenosine (m6A), 5-methyladenine (m5C), N1 methyladenosine (m1A), and 7-methyladenosine m7G methylation (5), have recently gained attention in epigenetic research. The m6A, m1A, and 5-m5C modifications are the most common in eukaryotic messenger RNA (mRNA) regulation. Current studies have proved that m6A, m1A, and m5C regulators play essential roles in methylation, which is related to tumor progression (6–9). M6A regulatory genes methyltransferase 3 (METTL3), METTL14, and WTAP reportedly initiated m6A modification (7). MRTTL3 is usually overexpressed in endometrial epithelial ovarian cancer (EEOC) and can be used as a risk factor for the overall survival of EEOC patients. Similarly, M5C methyltransferase NSUN2 is overexpressed in gastric cancer and can be used as a risk factor for the overall survival of gastric cancer patients. Cell experiments demonstrated that NSUN2 promoted gastric cancer cell proliferation, migration, and invasion (9). Several studies have recently developed genetic risk models to evaluate the prognostic status of cancer patients and demonstrated the independent roles of the predictive variables (10–13).

Researchers have found that although long non-coding RNA (lncRNA) cannot be converted to protein, it impacts many biological processes, such as tumorigenesis and progression (14, 15). Methylation-related lncRNAs are involved in various biological processes associated with cancer progression (15) and have recently been found to influence cell proliferation, migration, and metastasis of many tumors (16–18). Meanwhile, the relationship between methylation and lncRNAs is being extensively studied, but their interaction mechanism is still unclear.

The role of m6A, m1A, and m5C regulatory genes in the progression of low-grade gliomas needs to be better understood. Therefore, this study aimed to evaluate the biological roles of m6A/m1A/m5C regulatory genes in the progression of low-grade gliomas using data from the Cancer Genome Atlas (TCGA) database and identify the lncRNAs associated with their regulatory networks.

Currently, computational biology and high-throughput sequencing data have been widely used in the research of the biomedicine field by Yutao (10, 19, 20). Wang et al. used computational biology methods such as WGCNA to identify biomarkers in different tumors, which provided us with a reliable methodological basis for studying the mechanism of tumorigenesis (21, 22). Weighted gene co-expression network analysis (WGCNA) and a prognostic risk model were used to calculate the prognosis signature score for the low-grade gliomas with methylation-associated lncRNAs.

## Method

### Data collection

We accessed the TCGA database (https://portal.gdc.Cancer.gov/) to obtain the gene matrix profiles and the relevant clinical information of the low-grade glioma patients, including age, sex, survival time, survival rate, and tissue or organ sample availability. We obtained 514 low-grade glioma tumor samples from patients with primary tumors and metastatic gliomas (23), and 44 m6A/m5C/m1A regulators were determined based on the existing research on methylation (**Supplementary Table 1**). To ensure the accuracy and feasibility of analysis, we merged all data and converted them into TPM data format after downloading.

### Determination of methylation-related lncRNAs

We determined the lncRNA expression levels of the TCGA-low grade glioma (LGG) cohort and used Pearson’s correlation to identify 44 m6A/m5C/m1A methylation regulators associated with lncRNAs.

### LncRNA univariate COX regression analysis

We downloaded the clinical follow-up data, including disease status, of the TCGA-LGG cohort from the TCGA database and individually matched the gene expression data to the clinical information. We eliminated the samples with no match (20, 21, 24) and used univariate Cox proportional-hazards regression analysis to determine the lncRNAs highly associated with overall survival. The P < 0.05 indicated a significant prognostic statistical significance. These prognostic lncRNAs were used for non-negative matrix factorization and predictive model construction.

### Determination of the different lncRNA expression patterns related to methylation regulators

The prognostically significant lncRNAs were first clustered using non-negative matrix dimensionality reduction with 50 iterations. We obtained 9 clusters with the k-mer of 2-10, and the minimum sample size of each group was set to 10 by the ‘non-negative matrix factorization (NMF)’ R package. The number of our most desirable cluster groups was selected based on the Cophenetic, Dispersion, and Silhouette parameters. After that, survival analysis was used to determine the survival differences between the expression patterns, and P <0.05 was considered significant.

### WGCNA analysis

To investigate the biological differences among the different expression patterns of methylation-associated lncRNAs, we constructed protein-coding gene co-expression networks using the WGCNA method. We performed the functional enrichment of the co-expression networks. The TCGA-LGG co-expression network was created using the WGCNA R package, and optimum weighting parameters of the adjacent functions were obtained using the pickSoftThreshold function, which served as a soft threshold for subsequent network construction., Furthermore, the weighted adjacency matrix and the related gene modules were constructed based on the hierarchical clustering of the topological overlap matrix (25). To determine the biological significance of the co-expression modules, we calculated the correlation between the characteristic genes of each module and the NMF cluster analysis groups. Consequently, we identified the most relevant co-expression networks of methylation-associated lncRNAs.

### Intersection function analysis

The Database for The Annotation, Visualization, and Integrated Discovery (DAVID, v6.8) was used to annotate the protein-coding genes enriched in co-expression biology, biological processes, and cellular composition (26). Moreover, the Kyoto Encyclopedia of Genes and Genomes (KEGG) (https://www.genome.jp/kegg/) (27) and Gene Ontology (28) (http://geneontology.org/) analyses were applied to identify the biological function of the genes.

### Least absolute shrinkage and selection operator regression

The LASSO (29) regression algorithm was used to identify the prognostic survival of low-grade glioma patients and construct a predictive gene model. We used the single factor data of methylation-related lncRNAs to build the model, with the random number seed set as 27. After that, the time-dependent receiver operating characteristic curve (ROC) was used to evaluate the model’s predictive performance. The different survival outcomes between the two groups were compared using the Kaplan-Meier survival curve and the log-rank test.

### Immune microenvironment analysis

We assessed the proportion of immune cells in the immune microenvironment of TCGA-LGG using several methods. These methods included CIBERSORT (30, 31), EPIC (32), quanTIseq (33), MCPcounter (34), XCELL (35), and TIMER (36). After that, tumor purity of the tumor immune microenvironment was assessed using ESTIMATE, which estimated the proportion of stroma and immune cells in malignant tumor tissues using expression data to generate the purity score. The gene sets associated with multiple confirmatory responses were evaluated to explore the relationship between the model and the confirmatory responses in the immune microenvironment. These gene sets included major histocompatibility complex class II(MHC-II), lymphocyte-specific kinase (LCK), hematopoietic cell kinase (HCK), immunoglobulin G(IgG), signal transducer and activator of transcription 1(STAT1), costimulatory molecule (B7-CD28), interferon, and tumor necrosis factor (TNF) (37). Genes in these gene sets are presented in **Supplementary Table 2**.

## Results

### The research routine

**Figure 1:**
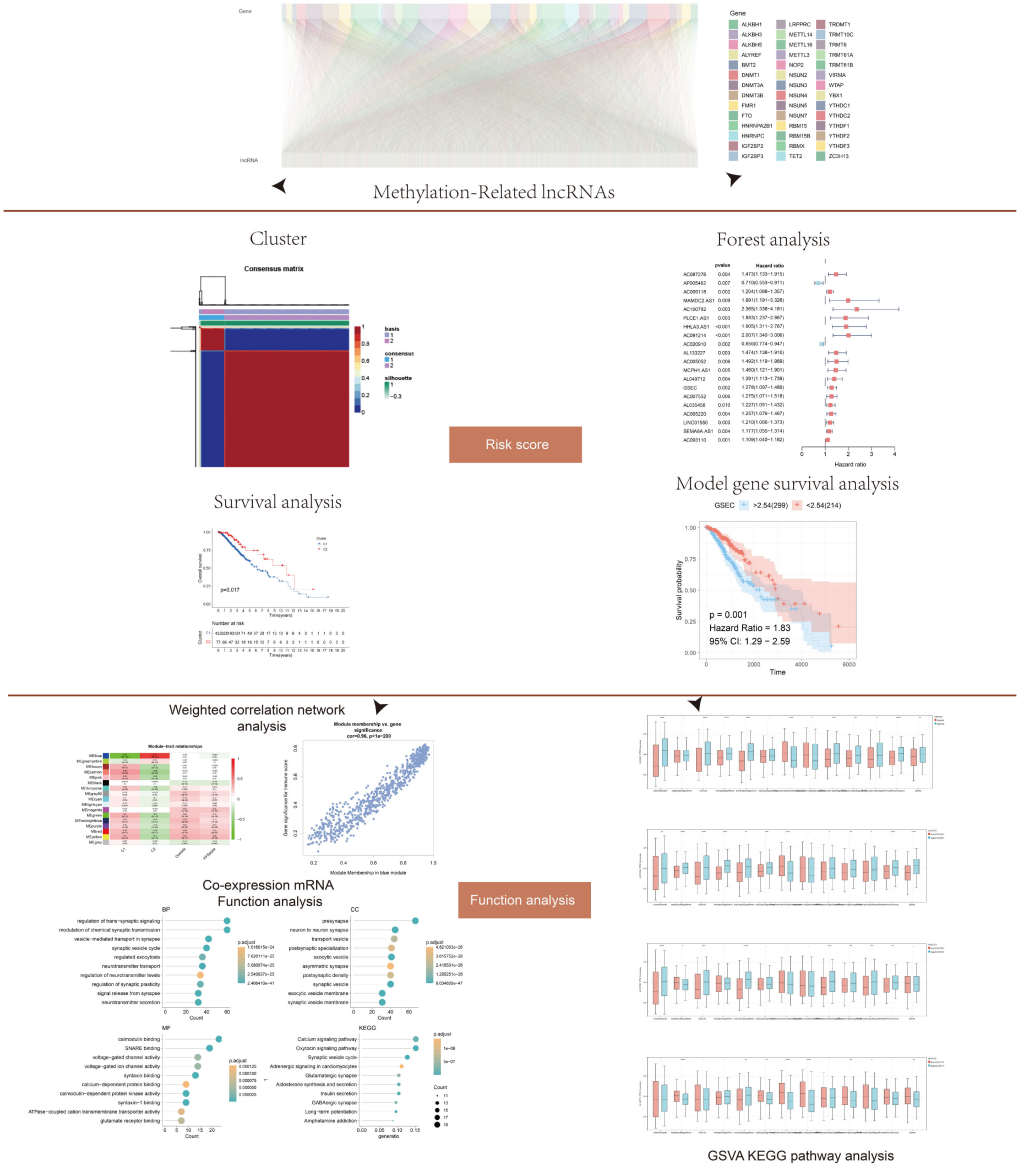
Flow chart showing the methodology of the study. Long non-coding RNAs (lncRNAs) associated with DNA methylation were screened from the literature. Cluster analysis, forest map, and survival analysis were used to determine the risk score. Functional analysis was performed *via* weighted gene co-expression network analysis (WGCNA), co-expression enrichment, and gene set variation analysis (GSVA) analyses. The model was verified by cell experiment.

Multiple methylation regulatory genes were obtained through a literature review using the analysis process shown in **Figure 1**. The Pearson correlation analysis identified the methylation-associated lncRNAs, and we subjected the lncRNAs with independent aftereffects to a prognostic analysis. Thus, two cohorts of low-grade gliomas with different expression patterns of lncRNA were obtained. The survival analysis revealed a significant difference in the overall survival between the two groups of patients with varying expression patterns. After functional enrichment, WGCNA was used to analyze the co-expression networks between the two groups and determine the differences in their biological functions. We also constructed a prognostic survival model for low-grade glioma using the lncRNAs to demonstrate the involvement of lncRNAs in cell proliferation and invasion through cell experiments.

**Figure 2:**
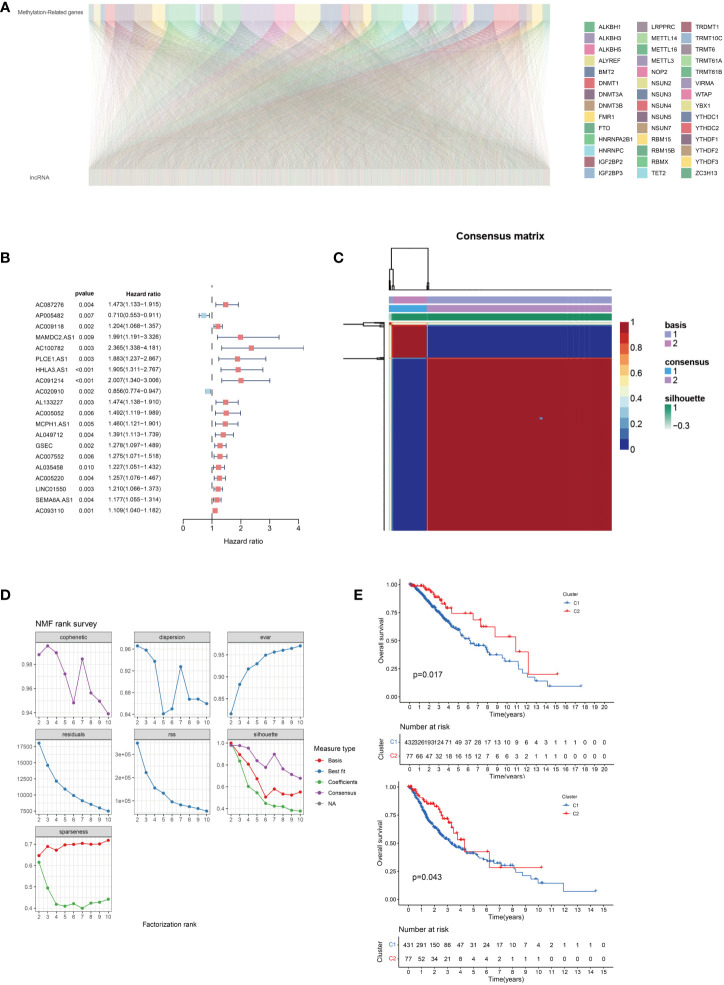
**(A)** Correlation between 5-methyladenine (m5C)-related genes and long non-coding RNAs (lncRNAs) in lower-grade gliomas. **(B)** Univariate Cox regression analysis of prognostic lncRNAs associated with m5C. **(C)** Negative matrix factorization clustering of m5C-relatedlncRNAs gene sets. **(D)** The parameters of negative matrix factorization clustering. **(E)** Overall and disease-free survival prognosis curve of the different subtypes.

We screened 2330 lncRNAs to identify those with correlation coefficients greater than 0.4 based on the 42 methylation-related protein-coding genes (**Figure 2A**). Using univariate COX regression, we analyzed the association between these lncRNAs and overall survival. The risk ratios of lncRNAs and the corresponding statistical parameters are shown in **Figure 2B**. AP005482 was a prognostic protective factor with a risk ratio of 0.710, and AC020910 was a prognostic protective factor for low-grade gliomas.

### Survival differences associated with the long non-coding RNA expression patterns

Cluster analysis of methylated lncRNAs was performed after the univariate COX regression analysis at P < 0.05. We used 108 lncRNAs for the non-negative matrix dimensionality reduction, whose results are shown in **Figures 2C, D**. There was a strong correlation between the two groups, as indicated by the red coloration; however, blue coloration indicated a weak correlation between the two groups. The clustering between the two groups was excellent, and there were no significant differences between the two groups. We also evaluated the overall and disease-free survival and found that the overall survival and relapse-free rates were lower in group C1 than in group red (**Figure 2E**).

### Identification of biological function differences between two different methylation patterns

**Figure 3:**
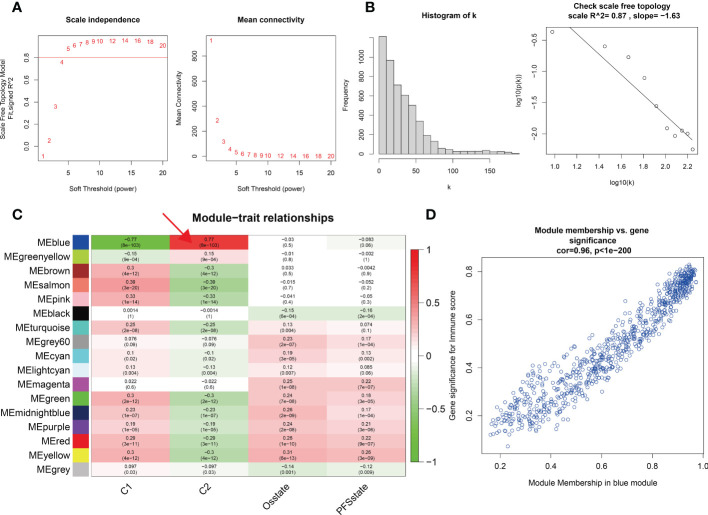
**(A, B)** Soft threshold and scale-free topology of weighted correlation network analysis. **(C)** Module-trait relationships of different modules and different molecular typing. The relationship between the blue module and C2 is mostly connective. **(D)** The Pearson correlation coefficient between the significance of the immune score and module membership in the blue module is 0.96.

**Figure 4:**
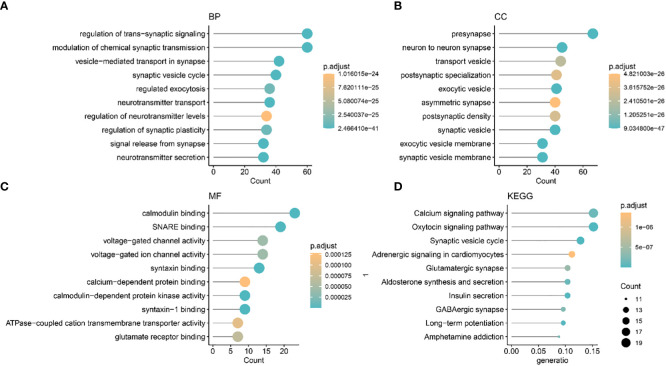
The Gene Ontology and Kyoto Encyclopedia of Genes and Genomes biological pathways showing the mainly enriched co-expression modules. **(A)** BP: biological process. **(B)** CC: Cellular component. **(C)** MF: Molecular function **(D)** KEGG.

We identified two different methylation-related expression patterns of the lncRNAs. WGCNA was used to analyze the protein-coding gene network of low-grade gliomas using the optimal soft threshold of 5 (**Figures 3A, B**). We obtained 17 co-expression modules which were then used to calculate the correlation of the different methylation expression patterns (**Figure 3C**). The correlation between the blue module and the C2 group was 0.77, while the correlation within the module was 0.96 (**Figure 3D**). We functionally enriched the blue module to determine the biological function differences between the different lncRNAs. We found that the co-expressed genes in the blue module were mostly related to trans-synaptic signaling in biological processes, modulation of chemical synaptic transmission, calmodulin binding, SNARE binding, calcium signaling pathway, and oxytocin signaling pathway (**Figure 4**).

### Screening and construction of long non-coding RNA prognostic models using machine learning methods

**Figure 5:**
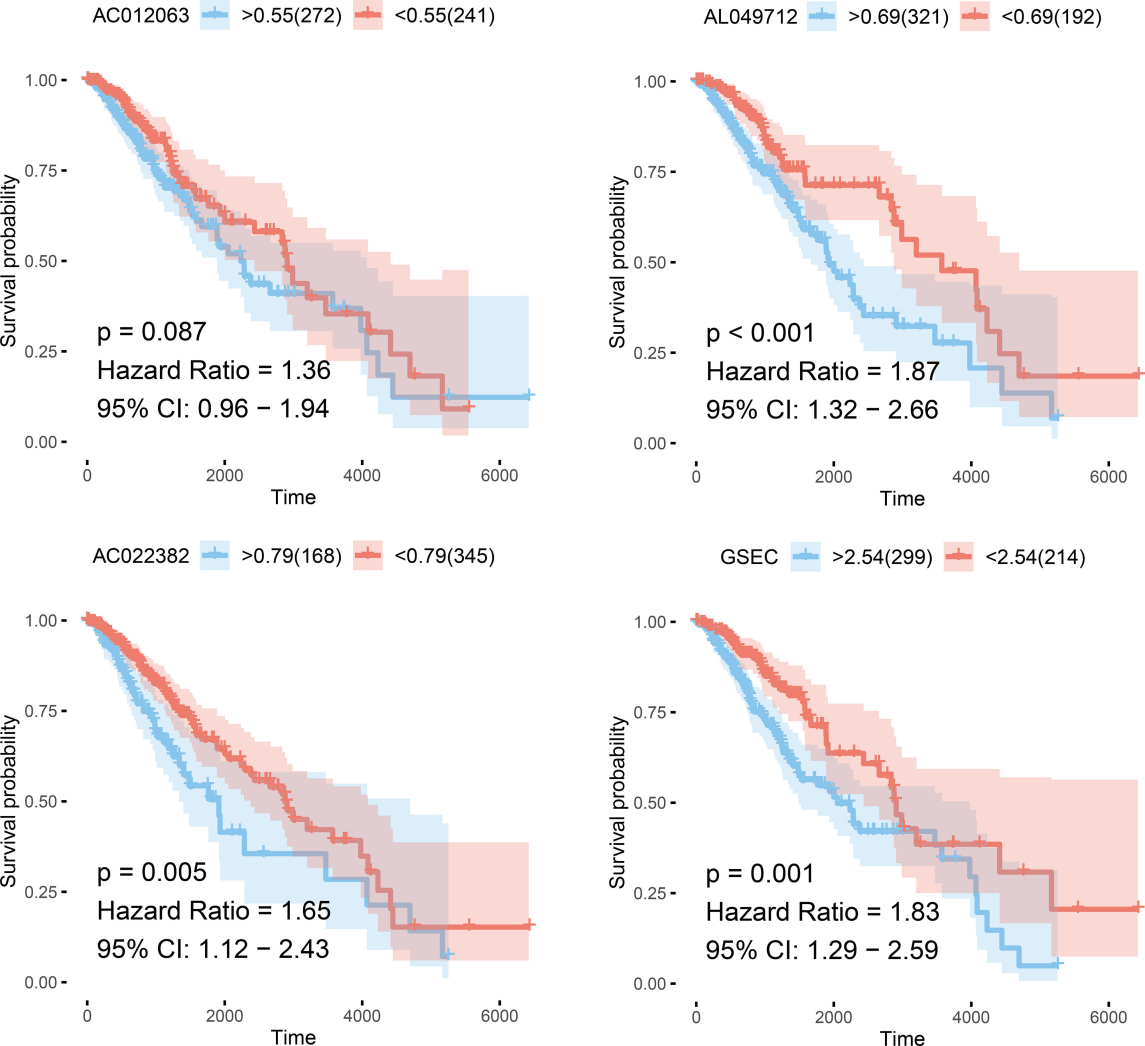
Survival curves of the four selected genes.

Using the random forest method, we first screened prognostic lncRNAs and identified 102 typical lncRNAs based on their importance ranking. LASSO regression analysis was then performed on these lncRNAs to construct a methylation-related prognostic model using four prognostically significant genes. The risk score of the model was 1.12 *AC012063 + 0.74 * AC022382 + 0.32 * AL049712 + 0.16 * GSEC. Furthermore, we analyzed the independent prognostic value of four lncRNAs in the prognostic model and found that the four lncRNAs encoded AC012063, AC022382, AL04971, and GSEC. All four lncRNAs were independent prognostic factors for low-grade gliomas (**Figure 5**).

### Result evaluation of the model

**Figure 6:**
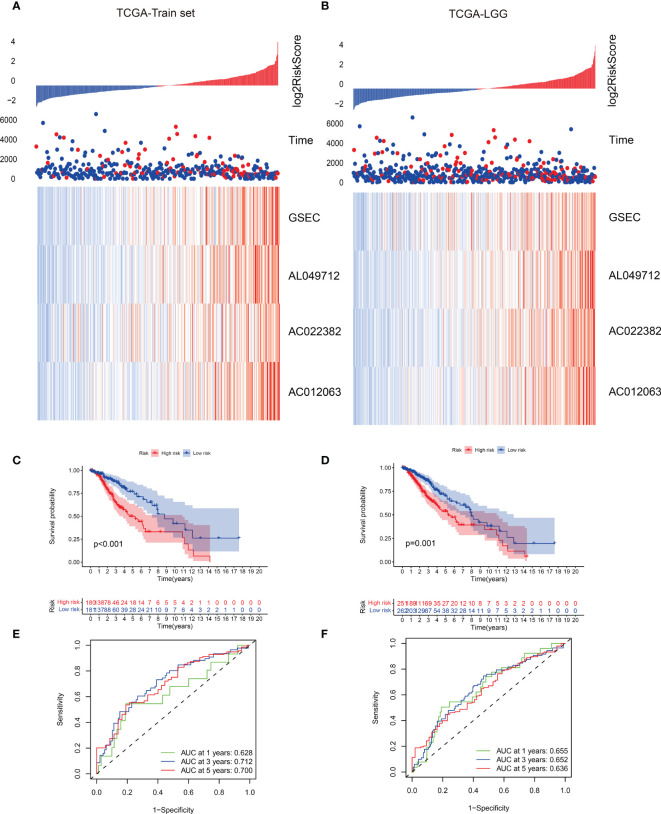
**(A, B)** Risk scores and survival status of gene signatures in the training and validation data set. **(C, D)** Survival curves of the two risk groups with different subtypes. **(E, F)** The receiver operating characteristic (ROC) curve of the two risk groups based on the gene signature classification.

We summarized the risk factor (gene) expression of each sample and the clinical follow-up information for generating a heatmap (**Figures 6A, B**). The samples were presented in ascending order in the heatmap based on their risk scores. Since patients with higher risk scores had a poor prognosis, we marked the actual survival status of the patients with red and blue plot points and determined the corresponding points on the ordinate survival time. The number of patients with red plot points increased, but their survival time reduced as the risk score increased. These plots were concentrated in the lower right corner of the heat map, demonstrating that patients with low-grade gliomas exhibit poor prognoses with increasing risk scores. This also indicated the possible prognostic roles of lncRNAs such as AC012063, AC022382, AL04971, and GSEC. Significant impact, suggesting important research value. The expression levels of the prognostic risk factors of each patient were annotated on the x-axis of the heatmap. The results showed that the expression levels of AC012063, AC022382, AL04971, and GSEC gradually increased with the risk score progression, but survival time reduced (**Figures 6A, B**). The survival curve and the ROC analysis results of the different risk groups were shown in **Figures 6C-F**, which indicated the patients with high risk score might lead the worse clinical outcome.

### LNCRNAs prognostic model and immune microenvironment and response

**Figure 7:**
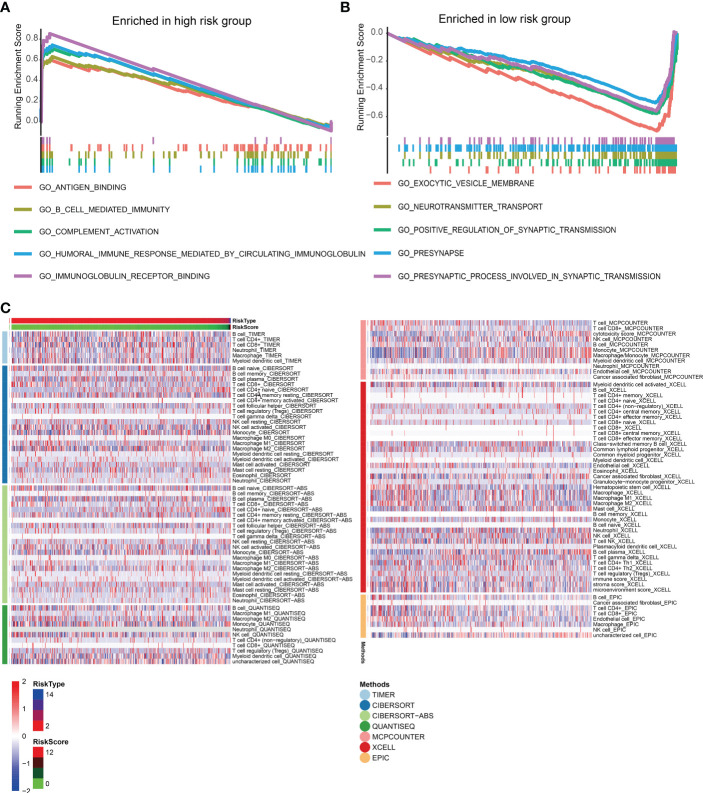
**(A, B)** The gene set enrichment analysis of high-risk and low-risk groups. **(C)** Heat maps of immune responses in the high-risk and low-risk groups based on TIMER, CIBERSORT, Cibersort-ABS, QUANTISEQ, MCPCOUNTER, XCELL, and EPIC algorithms.

The enrichment analysis showed that antigen binding, B cell-mediated immunity, complement activation, and immunoglobulin receptor binding were highly enriched in the high-risk score group. However, the low-risk group had significantly enriched exocytic vesicle membrane, neurotransmitter transport, and positive synaptic transmission regulation (**Figures 7A, B**). Based on these results, we further investigated how the risk scores related to the immune microenvironment and immune validation response. We assessed tumor purity and the immune and stromal scores in low-grade gliomas using the ESTIMATE method, and the analysis included 8 immune-validated response gene sets. The gene sets included virulent T lymphocyte-related biomarkers representing the strength of the cellular immune response. The immune-validation response gene sets, such as the tumor necrosis family, were also included. The results showed that IgG, HCK, MHC-II, LCK, STAT1 interferon, B7-CD28, and TNF-related tumor immune responses were significantly enhanced with the increasing risk scores, indicating that the immune microenvironment in high-risk glioma patients regulates response changes (**Figure 7C**).

### Effects of risk score-independent prognostic variables on biological pathways

**Figure 8:**
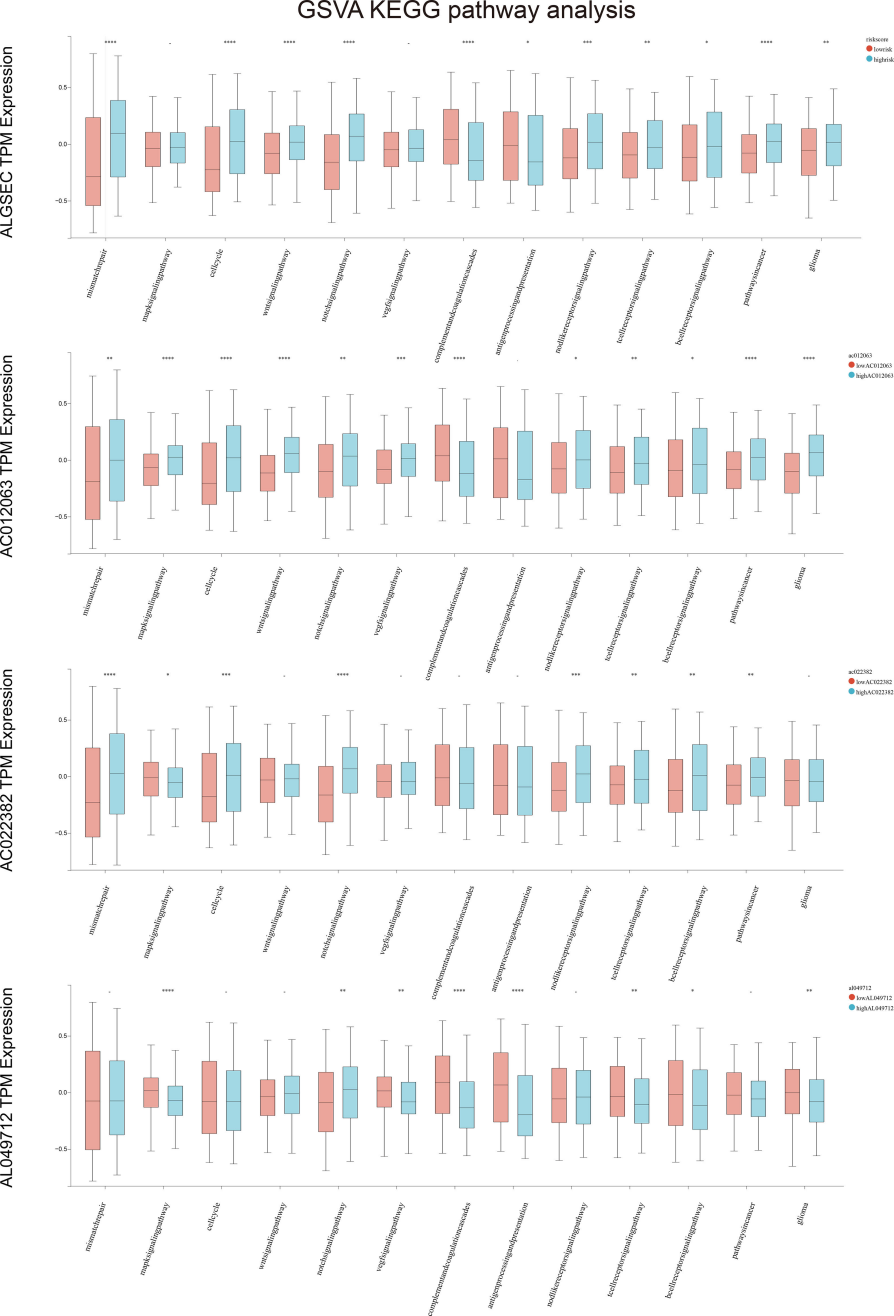
Gene set variation analysis (GSVA) in high and low-risk groups among four key genes. *p<0.05, **p<0.01, ***p<0.001, ****p<0.0001.

**Figure 9:**
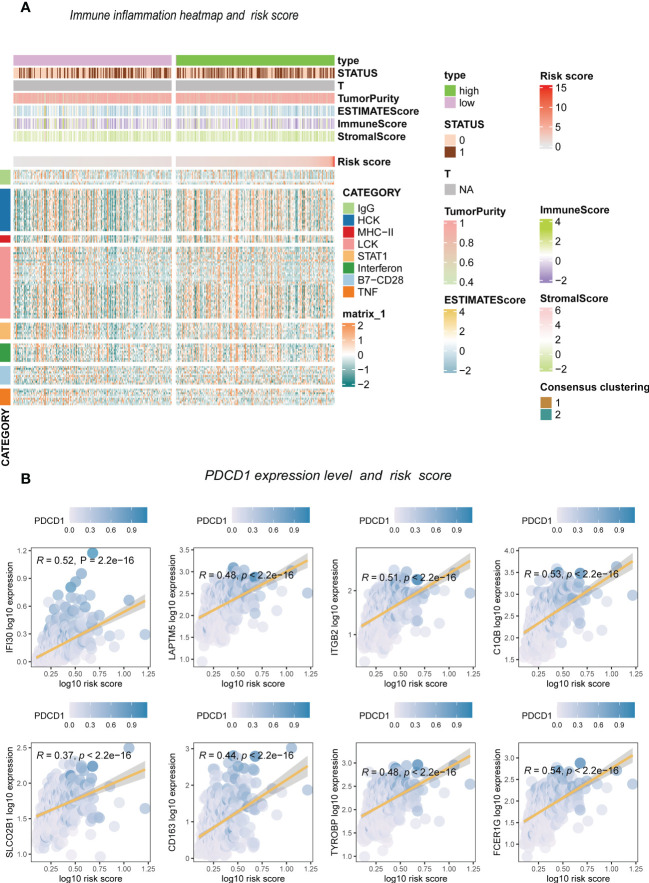
The inflammation response with risk score. **(A)** Heatmap. **(B)** Risk score and inflammation genes.

We divided patients into two groups based on the median expression of the risk score variables. We then assessed the gene set variation analysis (GSVA) score of the C2 KEGG pathway using the GSVA method and conducted t-tests with completely randomized data. The results showed that mismatch repair, cell cycle, wnt signaling pathway, NOTCH signaling pathway, complement, coagulation cascades, cancer pathways, and other pathways significantly differed in GSEC expression levels of the two groups. Groups with high GSEC expression were associated with poorer prognoses, and cell proliferation-related pathways, such as mismatch repair and cell cycle, were highly expressed in the group with high GSEC expression. Two classic biological pathways, WNT and NOTCH signaling, were also significantly upregulated. This suggests that GSEC may affect the prognosis of low-grade glioma cells by enhancing their proliferation, thus providing a reference for future research (**Figure 8**). In addition, we found higher levels of PDCD1 expression in groups with high risk scores (**Figure 9**).

## Discussion

Researchers are committed to developing prognostic assessment risk scores to evaluate cancer patients’ prognoses. However, the role of lncRNAs associated with methylation-related regulators in the prognosis of patients with low-grade gliomas and the immune microenvironment of malignant tumors is unclear. Given the heterogeneity of m6A/M1A/M5C methylation modifications in low-grade gliomas, it is essential to quantify the long-chain non-coding modification group in low-grade glioma patients. In low-grade gliomas, we identified many methylation-related regulators for screening and identifying methylation-associated lncRNAs. We identified methylation-related regulators that could be modified by lncRNAs from the literature and screened 2330 lncRNAs. Furthermore, four lncRNAs in LGG were used to establish a lncRNAs-based prognostic model to determine the overall survival and prognosis. Patients were divided into low-risk and high-risk groups according to their risk scores. The data showed that AC012063, AC022382, AL04971, and GSEC were prognostic lncRNAs associated with methylation regulators in LGG. Moreover, the AUC of the ROC curve showed that the methylation-associated lncRNAs prognostic model was more accurate than the ones reported in other studies due to its 5-year specific survival and specificity.

Methylation, a common epigenetic modification, plays a crucial role in gene expression regulation. Recent studies have revealed a complex interplay between DNA methylation and long noncoding RNA (lncRNA) in various biological processes. Specifically, lncRNAs have been shown to recruit DNA methyltransferases to specific genomic regions, leading to site-specific DNA methylation. Moreover, some lncRNAs have been found to function as “decoys” that prevent DNA methylation by sequestering DNA methyltransferases away from their target genes. In addition, lncRNAs themselves can also be subject to methylation, which affects their stability and expression levels. Thus, the relationship between methylation and lncRNA is intricate and multifaceted, and further research is needed to fully elucidate its mechanisms and biological implications.

Long non-coding RNA (lncRNA) has a variety of biological functions in glioma, including promoting or inhibiting tumor growth, metastasis, angiogenesis and drug resistance. Among them, lncRNA H19 has been extensively studied. H19, a hepatocyte growth factor (HGF) -induced lncRNA, has been shown to be highly expressed in a variety of tumors, including gliomas. H19 can promote the proliferation and self-renewal of glioma stem cells, and promote the migration and metastasis of tumor cells through different mechanisms, including the regulatory relationship with miRNA, EZH2-mediated epigenetic regulation, etc. Therefore, H19 may serve as a therapeutic target for glioma stem cells and tumor migration. Therefore, the use of computational biology in this study to analyze methylation-related long non-coding RNA is very important for the study of the genesis and development mechanism of glioma

In addition, lncRNA MALAT1 was also up-regulated in glioma. MALAT1 can regulate the proliferation, migration and invasion of tumor cells and participate in the malignant transformation of tumors. In addition, lncrnas such as CCAT1 and TUG1 also play a role in promoting tumor growth and metastasis in glioma. In general, lncrnas play an important role in the occurrence and development of glioma. Understanding their mechanism of action will help to discover new therapeutic targets and develop more effective therapeutic strategies. Overall survival in LGG could also be predicted by the methylation-associated lncRNAs prognostic model, suggesting its potential application in future clinical cohort studies on glioma. The role of DNA and epigenetic histone modifications in cancer progression has led to the development of various drugs, including histone deacetylase inhibitors and hypoxia-targeting drugs. However, studying the different methylation mechanisms in cells has recently gained attention.

m6A is one of the crucial post-transcriptional modifications of the protein-coding genes in cancer pathogenesis. However, the biological function of lncRNA methylation remains unclear. Several studies showed that m6A might be crucial in cancer pathogenesis, but the mechanisms by which lncRNAs influence cancer progression and metastasis are unclear. M6A modulators extensively modify lncRNAs to control gene expression and cell biology at the transcriptional and post-transcriptional levels. Zhang Jun et al. predicted the interaction between lncRNAs and alkylation repair homolog protein 5 (ALKBH5), a demethylase that reverses methylation. Furthermore, nuclear paraspeckle assembly transcript 1 (NEAT1) was evaluated by gene silencing, RT-PCR, nuclear and cytoplasmic separation, scraping test, and transwell migration test (38). Yewen Shi evaluated the biological function of hepatic nuclear factor 1α antisense RNA 1 (HNF1A−AS1) and its regulatory mechanism in laryngeal squamous cell carcinoma. The study found that HNF1A-AS1 may act as a tumor suppressor lncRNA in LSCC by regulating the epithelial-mesenchymal transition (EMT) process. As a result, new therapeutic targets and strategies were discovered for treating patients with nasopharyngeal carcinoma (NPC) (39). Overexpressing APCDD1L-AS1, a novel lncRNA, inhibited the growth and metastasis of ccRCC cells *in vitro* and *in vivo*. Dysregulation of histone expression caused by APCDD1L-AS1 overexpression may also inhibit ccRCC progression (40). However, APCDD1L-AS1 expression was decreased by DNA hypermethylation and inactivation of von Hippel Lindau (VHL) protein expression. METTL3-mediated modification upregulated LINC00958 by stabilizing its RNA transcript, and the LINC00958 activated miR-3619-5p to upregulate hepatoma-derived growth factor (HDGF) expression. This facilitated tumor lipogenesis and progression (41), indicating the importance of studying the methylation of lncRNAs.

We identified several lncRNAs that may be involved in methylation modification by analyzing the methylation-related regulator RNAs. G-quadruplex forming sequence containing (GSEC)-lncRNA is mostly associated with methylation in low-grade glioma but has also been extensively studied in various cancers. Jianhua Zhang et al. found that GSEC was significantly upregulated in TNBC tissues and cancer cell lines. Moreover, high GSEC levels were associated with tumor staging, positive lymph node metastasis, and poor prognosis in TNBC patients. The study also found that downregulating Mir-202-5p attenuated the inhibitory effect of GSEC knockdown on TNBC cell proliferation, invasion, and migration *in vitro*. Meanwhile, AXL overexpression reversed the *in vitro* mimicry inhibitory effect of Mir-202-5p on TNBC progression (42).

Shangshang Hu et al. constructed a GSEC/Mir-101-3p/SNX16/PAPOLG network to predict the prognosis of hepatocellular carcinoma (43). Xiulin Jiang et al. also found that ferroptosis-related GSEC-lncRNAs, mirNA-101-3p, and CISD1 axis play a functional role in lung adenocarcinoma (LUAD) and may serve as useful diagnostic and therapeutic biomarkers for the disease. The study reported that the ferroptosis-related GSEC- lncRNA/mirNA-101-3P/CISD1 axis could be an independent prognostic marker for lung adenocarcinoma (44).

The present study constructed a methylation-associated lncRNAs prognostic model using computational biology and public databases. The model proved accurate and reliable in training and validating data sets. This suggested the importance of the four long non-coding RNAs, and GSEC has been identified as a potential methylation-related lncRNA. Despite these findings, there were several limitations to this study. This study utilized information from public databases for the *in silico* analysis. Although we have proved the significance of GSEC in cancer progression through literature review, there is a need to verify these findings through more external cohorts and *in vivo* experiments.

## Conclusion

This study identified methylation-related lncRNAs in glioma and determined their expression patterns. We found two expression patterns of the methylation-related lncRNAs, and there was a significant difference between the two expression patterns. A prognostic model was also constructed based on these lncRNAs. GSEC was considered a lncRNA with a significant value in cancer progression, thus providing a basis for studying epigenetic methylation. Therefore, this study provides new strategies and research directions in the prognosis and treatment of glioma.

## Data availability statement

The original contributions presented in the study are included in the article/**Supplementary Material**. Further inquiries can be directed to the corresponding author.

## Ethics statement

The studies involving human participants were reviewed and approved by Affiliated Haikou Hospital of Xiangya School of South University. The patients/participants provided their written informed consent to participate in this study.

## Author contributions

YL: data analysis, methodology, figures construction, and article writing. YL, XL, and ZY: methodology and validation. YL, XL, and ZY: supervision. All authors contributed to the article and approved the submitted version.
